# Myelofibrosis and anemia: a German claims data analysis to describe treatment sequencing, survival outcomes, and healthcare resource utilization

**DOI:** 10.1007/s00277-026-07033-w

**Published:** 2026-06-04

**Authors:** Alexander Slowley, Tim d’Estrubé, Konstantin Neukirch, Scarlette Kienzle, Sophia Junker, Shiyuan Zhang, Thomas Wilke, Joachim R. Göthert

**Affiliations:** 1 GSK, Global Value, Evidence and Outcomes, London, UK; 2 GSK, Munich, Germany; 3 Ingress-Health HWM GmbH, a Cytel Company, Berlin, Germany; 4 GSK, Real-World Evidence & Health Outcomes Research, 1250 S. Collegeville Road, Collegeville, PA 19426–0989 USA; 5 IPAM e.V and AOK PLUS, Wismar, Germany; 6 Department of Hematology and Stem Cell Transplantation, West German Cancer Center (WTZ), University Hospital Essen, Essen, Germany

**Keywords:** Myelofibrosis, Anemia, Germany, Overall survival, Healthcare resource utilization, Treatment sequencing

## Abstract

Anemia presents a challenge in the management of myelofibrosis (MF). This symptom may be present at diagnosis or develop soon after, and around half of patients with MF require red blood cell transfusions within the first year. Progression to being transfusion dependent (TD) negatively affects prognosis and quality of life. This retrospective cohort study examined treatment sequencing from MF diagnosis, and overall survival (OS) and healthcare resource utilization (HCRU) from diagnosis and by transfusion status in newly diagnosed patients with MF in Germany. Data from 2010 to 2022 were collected from the AOK PLUS statutory health insurance fund database. Transfusion status was determined during a 180-day landmark period following first anemia treatment; patients were transfusion independent (TI) if they had received no transfusion, TD if they received ≥ 3 transfusions within 12 weeks, and transfusion requiring (TR) if they received transfusions but did not meet TD criteria during the landmark period. Of 555 patients assessed for treatment sequencing, only 41.8% received MF therapies (Janus kinase [JAK] inhibitors, peginterferon alpha-2a, immunomodulators, hydroxycarbamide, allogeneic hematopoietic stem cell transplantation), while 69.0% received anemia-directed interventions (59.8% being blood transfusions). Among patients receiving treatment for anemia (*n* = 233), transfusion need was associated with shorter median OS (TD: 14.9, TR: 33.3, and TI: 74.3 months after the landmark period) and higher HCRU burden. This study documented high clinical and economic burden of anemia in patients with MF within Germany, particularly among TD patients, highlighting the need for novel treatments providing better prevention of anemia in MF.

**Supplementary Information:**

The online version contains supplementary material available at https://doi.org/10.1007/s00277-026-07033-w.

## Introduction

Myelofibrosis (MF) is a rare hematological neoplasm characterized by clonal myeloproliferation, leading to the production of abnormal megakaryocytes and a buildup of fibrosis in the bone marrow, which impairs the normal production of blood cells [1–4]. This cancer is driven by genetic mutations in hematopoietic stem cells, the most common being Janus kinase (*JAK*) 2, calreticulin (*CALR*), and myeloproliferative leukemia virus (*MPL*) [1, 3, 4]. The clinical hallmarks of MF include splenomegaly, constitutional symptoms (fever, night sweats, and fatigue), severe anemia, thrombosis, bleeding, bone pain, and pruritus [1, 3].

Management of MF depends on the risk stratification and symptom burden of the patient [1, 5]. Allogeneic hematopoietic stem cell transplantation (allo-HSCT) is the only potential cure for MF, but its use relies on donor availability and is limited by the risk of life-threatening complications [1, 2, 6]. When allo-HSCT is not possible, treatment aims to control symptoms and complications and to reduce the risk of progression [2]. JAK inhibitors (JAKis) are a standard-of-care option for patients ineligible for allo-HSCT [1, 3]. However, issues of poor tolerability and drug resistance limit the use of particular JAKis, such as ruxolitinib or fedratinib, and anemia can also develop owing to their mechanism of action [5, 7, 8]. Momelotinib is a novel JAKi that was approved by the European Medicines Agency in January 2024 for patients with primary MF who have moderate-to-severe anemia [9]. Momelotinib has a different mechanism of action to that of previously available JAKis, and includes potent inhibition of type 1 kinase activin A receptor or activin receptor-like kinase-2 (ACVR1/ALK2). This unique mechanism of action stimulates erythropoiesis and, therefore, has a beneficial effect on anemia [10]. Momelotinib improves anemia in MF and prevents or reverses red blood cell transfusion dependence [10–13]. Other treatment options for MF include immunomodulators, interferon alpha, hydroxycarbamide/hydroxyurea, androgens, corticosteroids, and blood transfusions [1–3, 14].

Anemia represents a particular management challenge in MF [3, 14]. It is typically present at diagnosis in around one-third of patients and almost all patients become anemic over the course of their disease [3, 7, 15]. Moderate anemia in MF has previously been treated with supportive agents primarily to prevent worsening and progression to transfusion need [16]. However, supportive care may only provide transient benefit and the dose of first-line JAKis that have a negative impact on anemia may need to be lowered [16]. Regular blood transfusions are typically needed for severe anemia [16]. Around half of patients with MF require blood transfusions within 1 year of diagnosis, and many become dependent on regular transfusions [14, 15]. Importantly, progression to transfusion dependency negatively affects prognosis, reducing quality of life, worsening comorbidities, increasing the risk of leukemic transformation, and shortening survival [10, 17]. The impact of anemia and associated fatigue on patients is substantial and increases alongside worsening anemia severity [16]. Similarly, by worsening prognosis and quality of life, anemia also increases healthcare resource utilization (HCRU) and costs in patients with MF, causing increased hospital admissions, emergency department visits, and treatment expenditure [16].

Real-world data are needed to further understand the health and economic burden of anemia in MF. A retrospective, real-world, claims-based study in Germany explored anemia in patients first diagnosed with MF between 2010 and 2022 [18]. During this study period, ruxolitinib was the only JAKi available until fedratinib was approved in February 2021 [19, 20]. This study previously estimated the prevalence of MF in Germany to be 9.9–12.4 cases per 100,000 [18]. Among patients receiving treatment for MF, the data showed that approximately 50% required treatment for anemia. Furthermore, the need for anemia treatments and blood transfusions increased as the disease progressed and treatment experience grew, with rates of these interventions increasing with successive treatment lines [18]. In this article, we report further results from this study, assessing treatment sequencing, overall survival (OS), and HCRU in patients with MF, by transfusion status.

## Methods

### Study design

This was a retrospective, longitudinal cohort study of patients newly diagnosed with MF using anonymized claims data from AOK PLUS, a German statutory health insurance fund. The study period was from January 1, 2010, to December 31, 2022, and the index date was the date of MF diagnosis.

The objectives were to assess real-world treatment sequencing in newly diagnosed patients with MF, in addition to OS and HCRU from MF diagnosis and according to transfusion status (transfusion-independent [TI], transfusion-dependent [TD], and transfusion-requiring [TR]).

### Data source

In 2021, the AOK PLUS fund covered approximately 3.3 million people, representing 54% of the local population in Saxony and Thuringia and 4.5% of the statutorily insured population in Germany [18, 21]. The analysis used anonymized, retrospective, and non-interventional data, so neither ethical approval nor patient informed consent were required [22].

### Study population

Adult patients were included if they had at least one inpatient diagnosis of MF or two outpatient MF diagnoses in different quarters within 1 year from January 1, 2012, to December 31, 2021; at least one inpatient or confirmed outpatient diagnosis of osteomyelofibrosis or at least one prescription or inpatient administration of a JAKi following the first observed MF diagnosis; and were continuously enrolled with the health insurance fund for at least 365 days prior to index diagnosis (see also Supplementary Methods and Table S1).

### Analysis cohorts

Analyses were conducted on three cohorts. These comprised a treatment sequencing cohort (to evaluate treatment patterns), an OS/HCRU descriptive cohort (to describe OS and HCRU from the MF index date), and a transfusion status cohort comprising patients receiving treatment for anemia (to evaluate OS and HCRU by transfusion status). For additional details, see Supplementary Methods.

### Identification of transfusion status

Transfusion status was defined for patients meeting the eligibility criteria of the transfusion status cohort and was determined during a 180-day landmark period following the first anemia treatment. Patients were categorized as TI if they had received no transfusion, TD if they received ≥ 3 transfusions within 12 weeks and TR if they received transfusions but did not meet TD criteria during the landmark period. Among TD patients, the date of their third transfusion marked the start of dependency.

### Assessments

Demographics and baseline clinical characteristics were assessed in each cohort. Real-world treatment sequencing (treatment rate, time to treatment initiation and discontinuation/switch, and time to TD) was assessed in the treatment sequencing cohort, which comprised newly diagnosed patients with MF. OS was measured from the index date (i.e., from MF diagnosis) in the OS/HCRU descriptive cohort. OS was also assessed by transfusion status from the landmark date (i.e., the last day of the 180-day landmark period following first anemia treatment) in the transfusion status cohort. HCRU was evaluated from the index date in the OS/HCRU descriptive cohort and by transfusion status from the landmark date in the transfusion cohort (Supplementary Methods, Table S2 and Table S3).

### Statistical analysis

See Supplementary Methods.

## Results

A total of 4,155 patients were identified with a diagnosis of MF from January 1, 2012, to December 31, 2021. Following exclusions, 555 patients were included in the treatment sequencing cohort and 510 patients in the OS/HCRU descriptive cohort (Fig. S1). A total of 233 patients were included in the transfusion status cohort for analysis of OS and HCRU by transfusion status, of whom 60 were TI, 109 were TR, and 64 were TD.

A total of 228 patients were successfully matched on propensity-score matching (173 non-TI patients [i.e. TD or TR] and 55 TI patients) and, after 1:2 nearest-neighbor matching with replacement, the matched cohort had a total of 346 patients (173 non-TI patients and 173 TI patients).

### Demographics and baseline clinical characteristics

The mean age of patients within the transfusion status cohort (*n* = 233) was 73.3 years and 57.9% were male (Table 1). Compared with TI patients, TR and TD patients tended to be older (TI, 69.6 years; TR, 74.6 years; and TD, 74.4 years), had higher comorbidity scores (TI, 4.7; TR, 5.2; and TD, 6.0), and more frequently had diagnoses of thrombocytopenia (TI, 18.3%; TR, 24.8%; and TD, 50.0%). Mean follow-up was 29.9 months.

**Table 1:** Demographics and baseline characteristics by transfusion status (transfusion status cohort)

Characteristic	All (*n* = 233)	TI (*n* = 60)	TR (*n* = 109)	TD (*n* = 64)
Patient demographics				
Age at landmark date, mean (SD)	73.3 (12.1)	69.6 (13.1)	74.6 (11.1)	74.4 (12.2)
Sex, male, *n* (%)	135 (57.9)	39 (65.0)	54 (49.5)	42 (65.6)
Landmark date year, *n* (%)				
2012–2014	60 (25.8)	11 (18.3)	24 (22.0)	25 (39.1)
2015–2017	64 (27.5)	15 (25.0)	31 (28.4)	18 (28.1)
2018–2020	73 (31.3)	19 (31.7)	37 (33.9)	17 (26.6)
2021–2022	36 (15.5)	15 (25.0)	17 (15.6)	4 (6.3)
Comorbidities (365 days prior to landmark date to 1 day prior to landmark date)				
Charlson Comorbidity Index score, mean (SD)^a^	5.3 (3.3)	4.7 (3.4)	5.2 (3.1)	6.0 (3.6)
Charlson Comorbidity Index score category, *n* (%)^a^				
0–1	31 (13.3)	12 (20.0)	14 (12.8)	5 (7.8)
2–3	52 (22.3)	15 (25.0)	22 (20.2)	15 (23.4)
4–5	47 (20.2)	10 (16.7)	23 (21.1)	14 (21.9)
6+	103 (44.2)	23 (38.3)	50 (45.9)	30 (46.9)
Anemia diagnosis, *n* (%)	203 (87.1)	37 (61.7)	105 (96.3)	61 (95.3)
Thrombocytopenia, *n* (%)	70 (30.0)	11 (18.3)	27 (24.8)	32 (50.0)
Essential thrombocythemia, *n* (%)	2 (0.9)	0 (0.0)	1 (0.9)	1 (1.6)
Polycythemia vera, *n* (%)	0 (0.0)	0 (0.0)	0 (0.0)	0 (0.0)
Baseline healthcare resource utilization (365 days prior to landmark date to 1 day prior to landmark date)				
All-cause hospitalizations, *n* (%)	210 (90.1)	52 (86.7)	99 (90.8)	59 (92.2)
Number of events, mean (SD)	4.9 (5.8)	3.8 (3.9)	4.9 (6.0)	5.9 (6.7)
Oncologist/hematologist visits, *n* (%)	146 (62.7)	38 (63.3)	62 (56.9)	46 (71.9)
Number of events, mean (SD)	6.7 (9.4)	6.7 (9.4)	4.7 (7.7)	10.3 (11.0)
Follow-up (landmark date to end of follow-up or censor)				
Follow-up time (months), mean (SD)	29.9 (26.3)	34.3 (23.3)	31.6 (26.5)	22.8 (22.8)
Acute myeloid leukemia transformation during follow-up, *n* (%)	10 (4.3)	0 (0.0)	6 (5.5)	4 (6.3)

*SD* standard deviation, *TD* transfusion-dependent, *TI* transfusion-independent, *TR* transfusion-requiring

^a^unadjusted for age

Baseline characteristics for the treatment sequencing and OS/HCRU descriptive cohorts, plus the matched cohort, are summarized in Table S4. Patients in the treatment sequencing and OS/HCRU descriptive cohorts had fewer comorbidities, less pre-existing anemia, and lower baseline rates of HCRU than the transfusion cohort.

### Treatment sequencing

#### MF treatment

Of the 555 patients assessed for treatment sequencing, 39.6% of patients had received treatment for MF within 4 years of diagnosis, and only 41.8% by the end of follow-up. Among the 232 patients who received treatment for MF during follow-up, the majority were treated within the first 6 months after MF index (*n* = 150, 64.7%). The most common treatments for MF were ruxolitinib (28.6%) and hydroxycarbamide (20.4%), with few patients receiving allo-HSCT (4.3%) throughout follow-up.

Among the 232 patients receiving a first-line (1 L) treatment, 24.6% received 1 L treatment until lost to follow-up, 22.0% died during 1 L treatment, 8.2% died after discontinuing 1 L treatment, 6.5% discontinued 1 L treatment and were lost to follow-up, and 2.6% received allo-HSCT and no further treatment thereafter. A total of 148 patients receiving 1 L treatment (63.8%) did not receive a further treatment line.

Among the 84 patients who received second-line (2 L) treatment, 24 (28.6%) advanced to third-line (3 L), 25.0% continued with 2 L treatment, 16.7% discontinued 2 L treatment (6.0% were lost to follow-up and 10.7% died during follow-up), 9.5% received no further treatment after allo-HSCT, and 23.3% died during 2 L treatment.

**Fig. 1:**
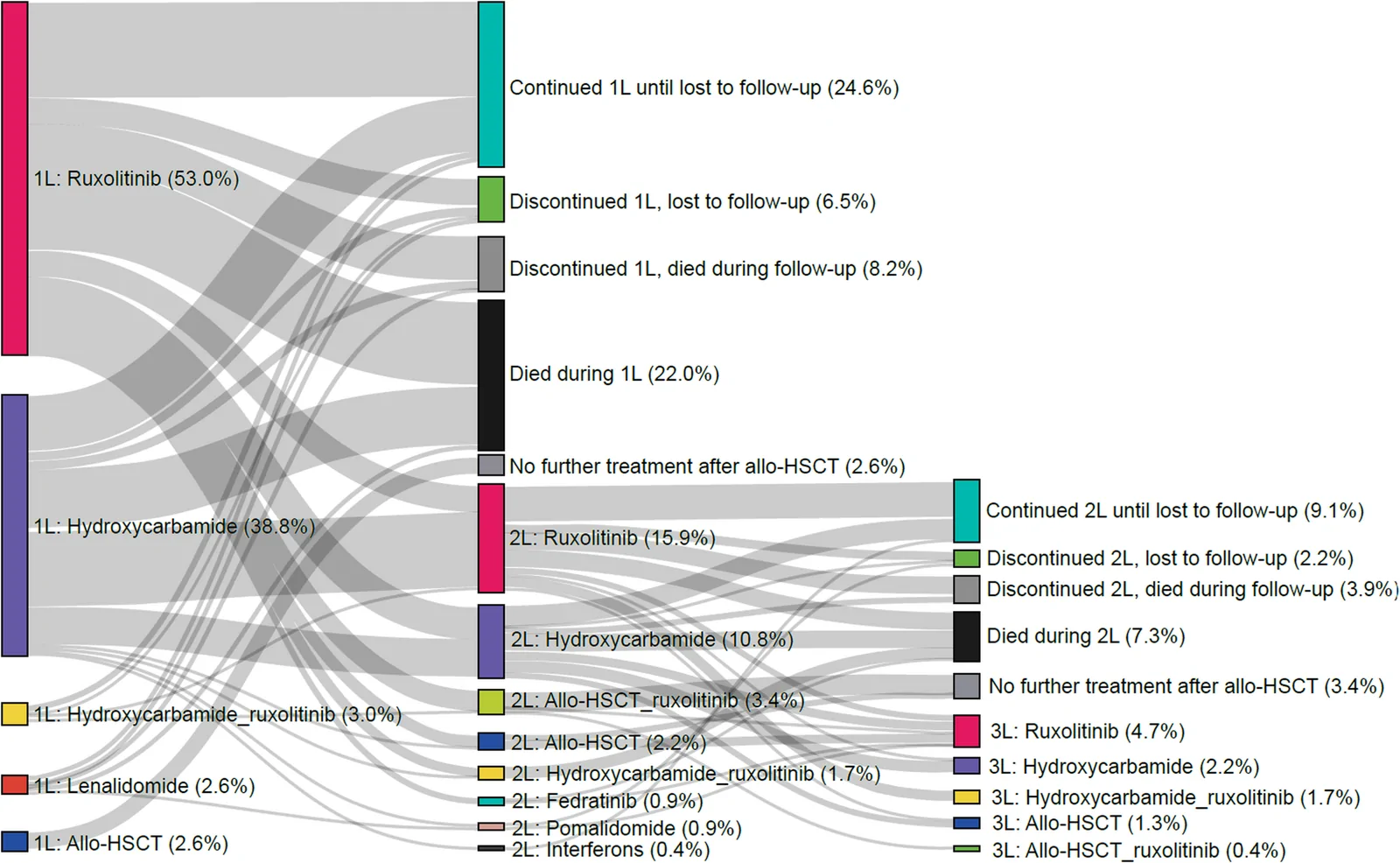
MF treatment regimens by line of therapy in patients receiving at least one treatment (treatment sequencing cohort *n* = 232). Note that fedratinib was only available following its approval by the European Medicines Agency in February 2021. 1L: first-line; 2L: second-line; 3L: third-line; allo-HSCT: allogeneic hematopoietic stem cell transplantation; MF: myelofibrosis

Figure 1 summarizes the MF treatment sequencing observed from 1 L to 3 L in patients receiving at least one treatment. Ruxolitinib was the most common 1 L treatment (*n* = 123/232, 53.0%, noting limited availability of other JAKis at the time of the study), followed by hydroxycarbamide (*n* = 90, 38.8%). Few patients received both ruxolitinib and hydroxycarbamide in combination (3.0%), lenalidomide (2.6%), or allo-HSCT (2.6%). Ruxolitinib and hydroxycarbamide were also the most common treatments in 2 L and 3 L (ruxolitinib 2 L: 44.0%, 3 L: 45.8%; hydroxycarbamide 2 L: 29.8%, 3 L: 20.8%).

Among patients who received ruxolitinib in 1 L, 26.8% continued ruxolitinib until censored, 19.5% discontinued, 23.6% died during 1 L, 22.8% switched to another treatment (12.2% to hydroxycarbamide [with or without ruxolitinib], 8.9% to allo-HSCT [with or without ruxolitinib], and 1.6% to fedratinib), and 7.3% discontinued ruxolitinib and then later resumed treatment.

#### Concomitant treatment (including anemia treatment/transfusions)

**Fig. 2:**
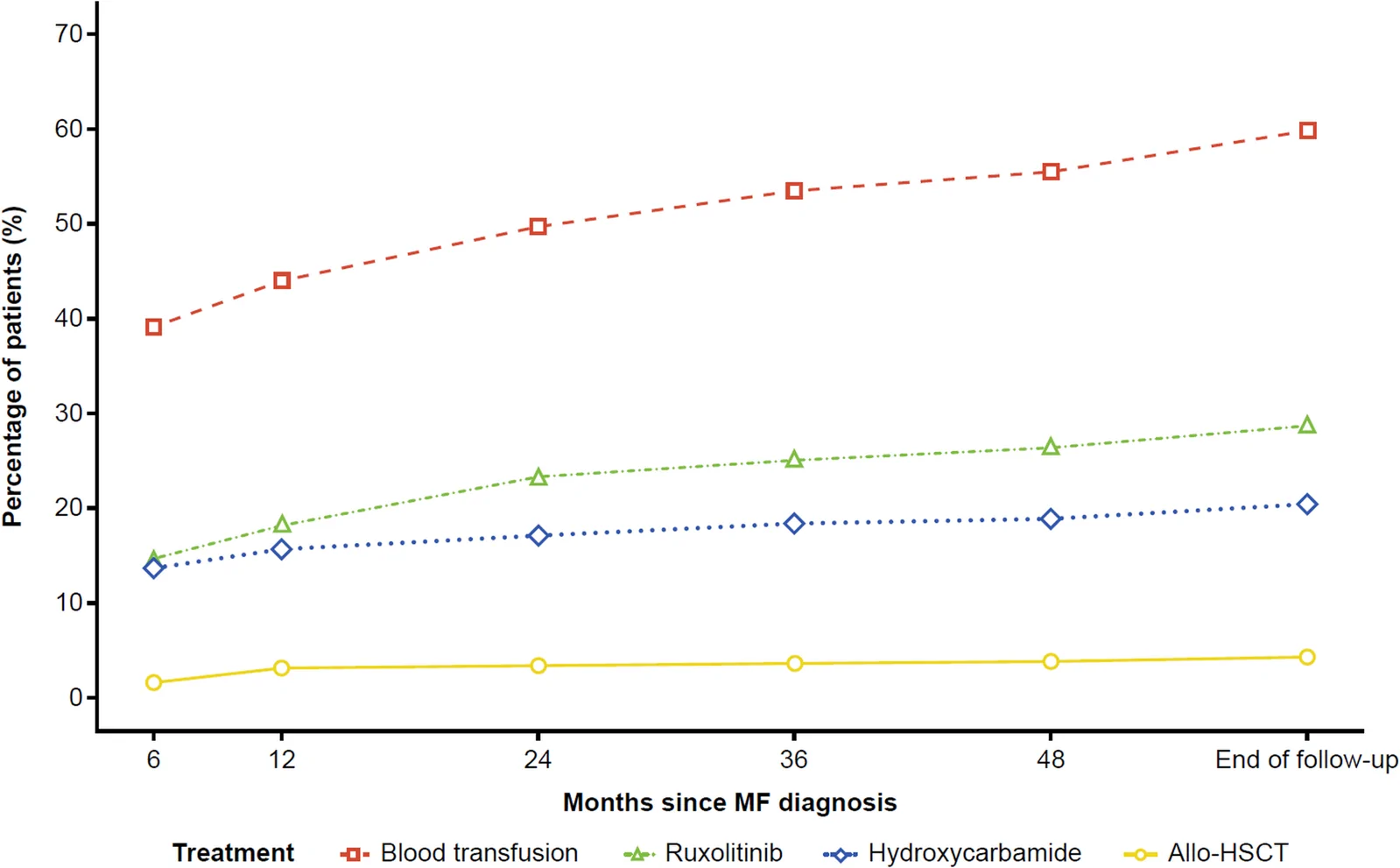
Cumulative percentage of patients who received key MF or concomitant treatments over time (treatment sequencing cohort *n* = 555), allo-HSCT: allogeneic hematopoietic stem cell transplantation; MF: myelofibrosis

Among the 555 patients assessed for treatment sequencing, concomitant treatments were given to 69.4% of patients, with 69.0% of patients receiving treatment for anemia. This intervention comprised blood transfusions (59.8%), corticosteroids (32.1%), erythropoietin (4.3%), and androgens (0.2%). More patients received blood transfusions during follow-up than ruxolitinib, hydroxycarbamide, or allo-HSCT (Fig. 2).

#### Time to treatment initiation and discontinuation/switch

The median time to initiation of treatment for MF was 3.1 months after diagnosis (*n* = 232). Among patients who received a JAKi, median time to treatment initiation was 5.6 months after MF diagnosis (*n* = 159). Patients receiving anemia treatment did so shortly after MF diagnosis (median time to initiation of 1.6 months, *n* = 383), earlier than MF treatment.

**Fig. 3:**
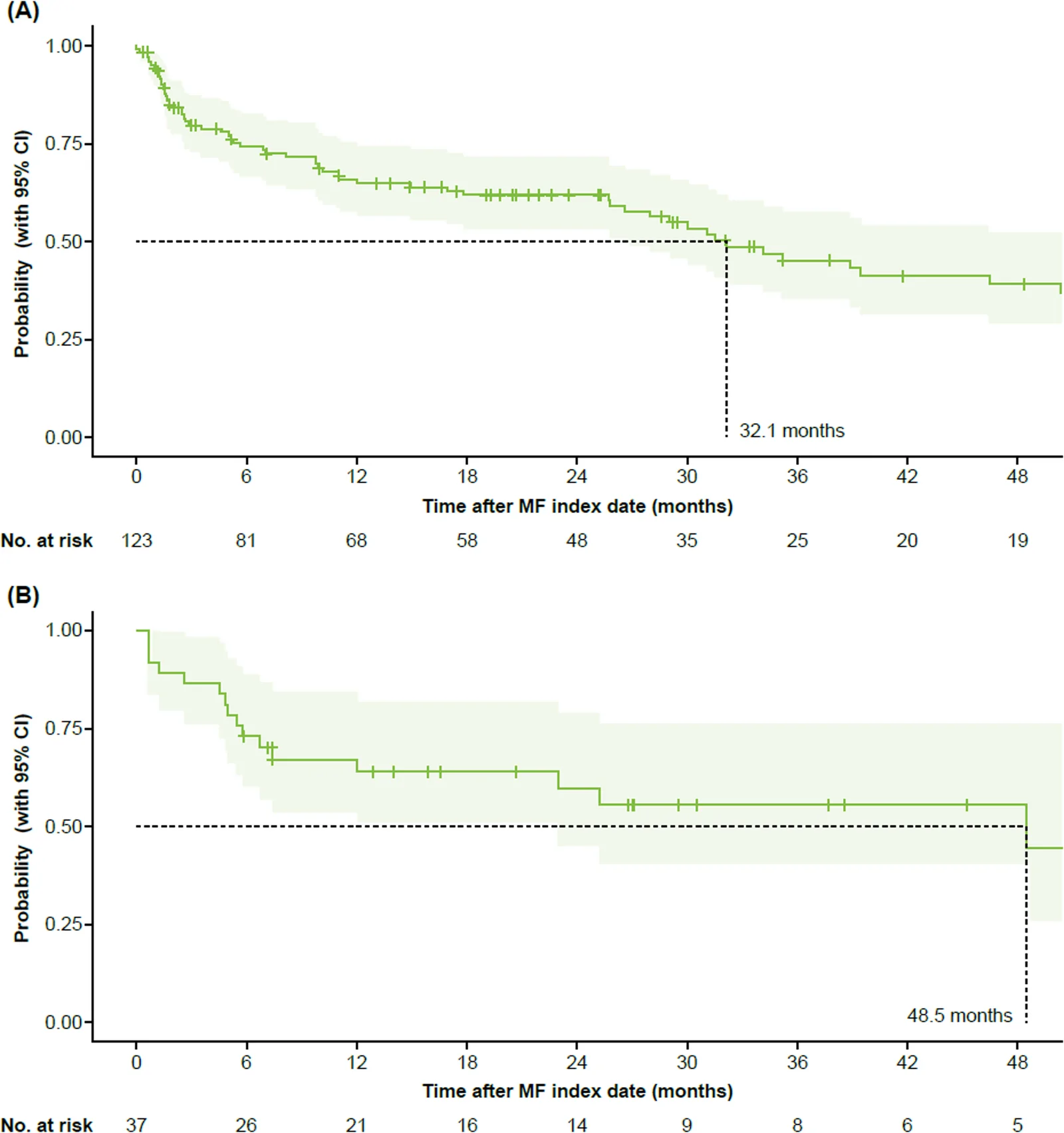
Time to ruxolitinib treatment discontinuation or switch in patients receiving ruxolitinib in (**A**) 1 L treatment (*n* = 123) and (**B**) 2 L treatment (*n* = 37). 1L: first-line; 2L: second-line; CI: confidence interval; MF: myelofibrosis

Time to treatment discontinuation/switch was longest for 1 L (median 28.4 months) and was shorter for later treatment lines (2 L, median 14.1 months; 3 L, median 4.9 months). For patients receiving ruxolitinib, the median time to treatment discontinuation/switch was 32.1 months after MF index date in the 1 L setting (*n* = 123; 64 patients discontinued/switched) and 48.5 months after MF index date in the 2 L setting (*n* = 37; 18 patients discontinued/switched; Fig. 3).

#### Time to transfusion dependency

**Fig. 4:**
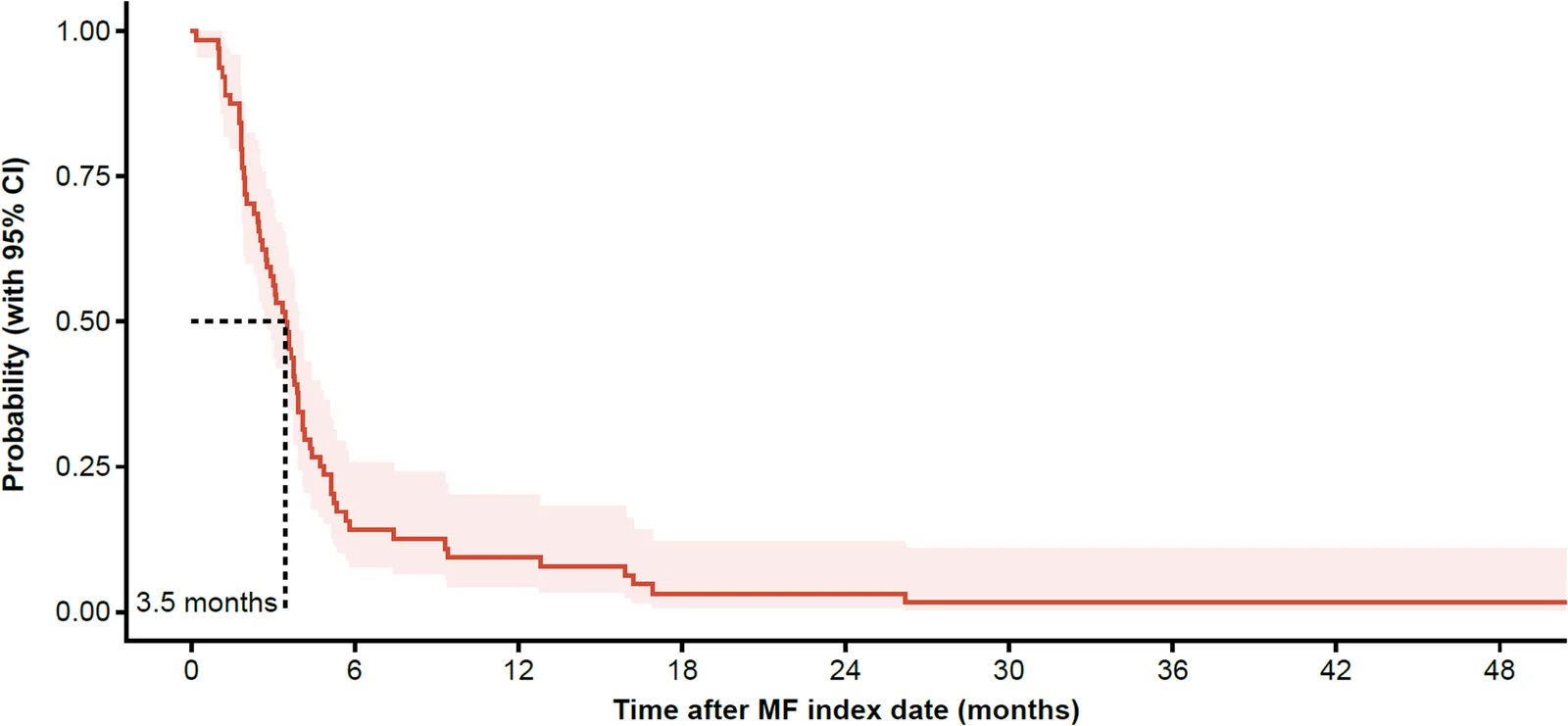
Time to transfusion dependency among transfusion-dependent patients (*n* = 64). CI: confidence interval; MF: myelofibrosis

Among TD patients (*n* = 64; i.e., patients receiving a third transfusion within 12 weeks during the 180-day landmark period following the first anemia treatment), 85.9% became dependent on transfusions within 6 months of MF diagnosis. This proportion increased to 90.6% and 96.9% at 12 and 24 months following MF diagnosis, respectively. The median time to transfusion dependency was 3.5 months (95% confidence interval [CI] 2.7–3.9; Fig. 4).

#### OS

The median OS from MF index date in the OS/HCRU descriptive cohort was 55.4 months (95% CI 46.5–67.1). Among patients receiving anemia treatment and who were assessed for transfusion status (transfusion status cohort), median OS from MF diagnosis was 44.6 months.

**Fig. 5:**
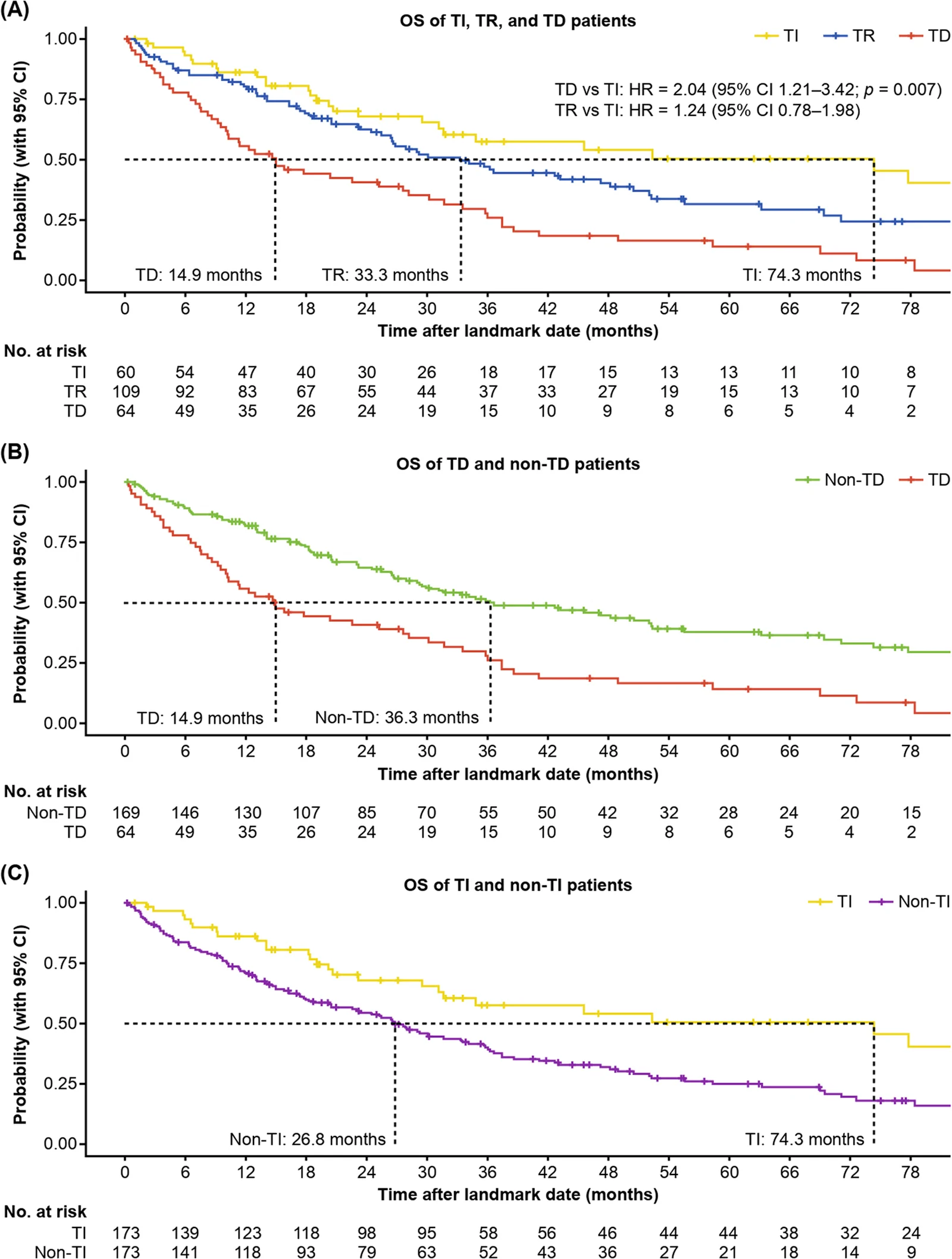
Overall survival by transfusion status (transfusion status cohort; *n* = 233). CI: confidence interval; HR: hazard ratio: OS: overall survival; TD: transfusion-dependent; TI: transfusion-independent; TR: transfusion-requiring

Transfusion need was associated with shorter median OS rates. The median OS after the landmark date was longest for the TI group (74.3 months), followed by the TR group (33.3 months), and was shortest for the TD group (14.9 months; Fig. 5A). Using unmatched data, transfusion status of TD compared with TI significantly reduced OS (HR = 2.04; 95% CI 1.21–3.42; *p* = 0.007), while the HR for TR versus TI transfusion status was not statistically significant (HR = 1.24; 95% CI 0.78–1.98). Both analyses directionally favored TI in terms of OS over the other group.

Across all non-TD patients (i.e., TI and TR patients), median OS after the landmark date was 36.3 months, which was again longer than OS for patients who were TD (Fig. 5B). Conversely, among all non-TI patients (i.e., TD or TR), median OS after the landmark date was 26.8 months (Fig. 5C). Mortality rates per patient-year of follow-up were 0.16 in TI patients versus 0.30 in non-TI patients.

When comparing TI and non-TI patients, transfusion status did not significantly affect OS both before matching in a multivariate Cox regression model (HR = 1.46; 95% CI 0.95–2.26) or after propensity-score matching (HR = 1.23; 95% CI 0.43–2.13). Using unmatched data, other covariates that had a significant effect on survival when comparing TI and non-TI patients were higher age (HR = 1.06; 95% CI 1.03–1.08; *p* < 0.001), male sex (HR = 1.67; 95% CI 1.15–2.44; *p* = 0.008), and presence of thrombotic disease (HR = 1.50; 95% CI 1.05–2.15; *p* = 0.026).

#### HCRU

HCRU from the MF index date in the OS/HCRU descriptive cohort is described in Table 2. The mean all-cause and MF-specific hospitalization rates were 5.0 and 0.9, respectively.

**Table 2:** Healthcare resource utilization from MF index date (OS/HCRU descriptive cohort)

	OS/HCRU descriptive cohort (*n* = 510)
Hospitalizations (from MF index date), mean (SD)	
All-cause hospitalizations	5.0 (5.9)
Hospitalization days (all-cause)	41.2 (52.1)
MF-specific hospitalizations	0.9 (3.0)
Hospitalization days (MF-specific)	6.5 (16.7)
Outpatient visits (from MF index date), mean (SD)	
Specialist visits (oncologist or hematologist)	15.0 (26.9)
General practitioner visits	39.1 (36.3)
Transfusions (from MF index date), mean (SD)	
Inpatient visits with associated transfusion	1.8 (3.6)
Outpatient visits with associated transfusion	4.1 (13.3)
Total number of blood transfusion procedures	9.7 (26.2)

*HCRU* healthcare resource utilization, *MF* myelofibrosis, *OS* overall survival, *SD* standard deviation

**Fig. 6:**
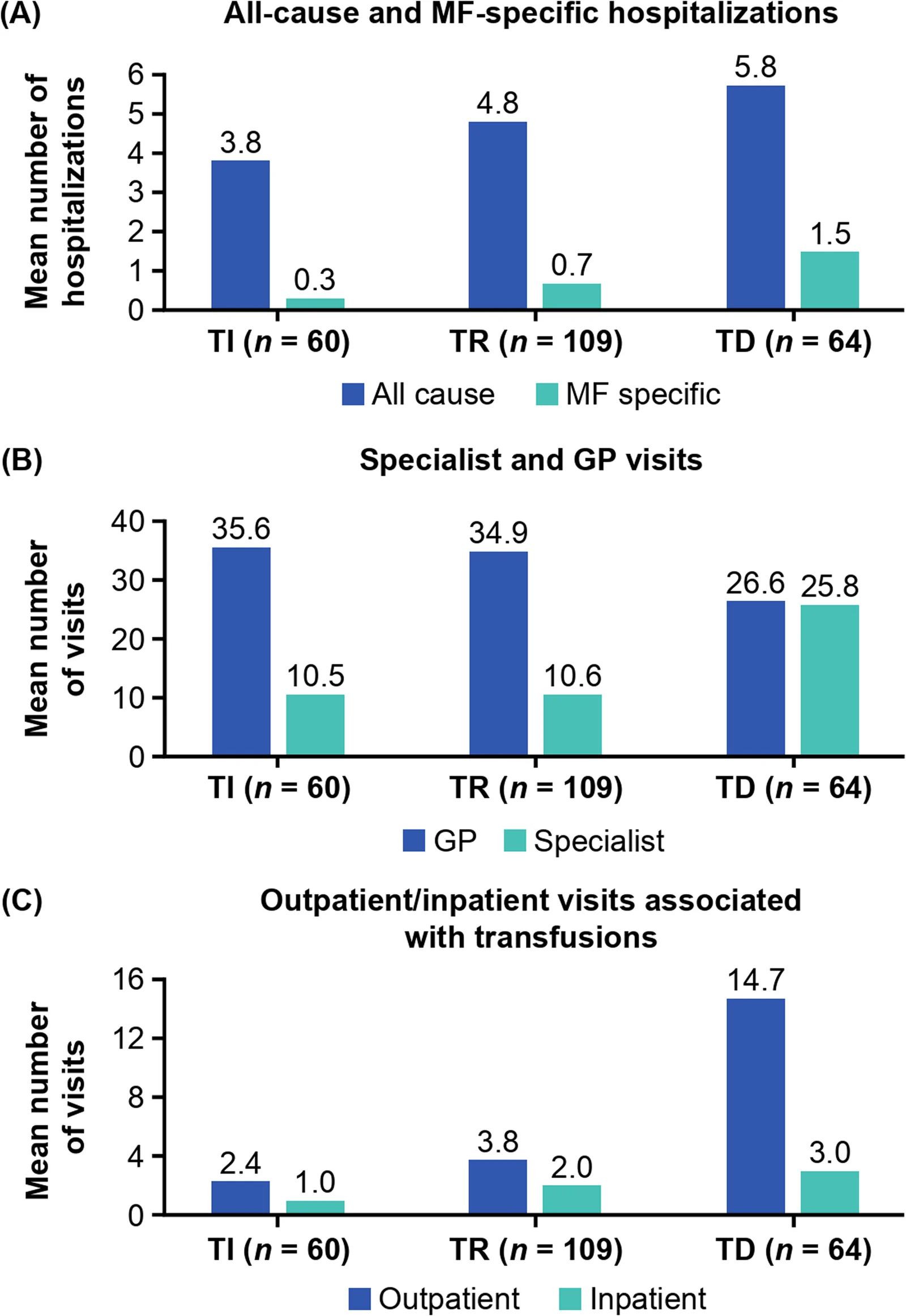
Healthcare resource utilization from landmark date by transfusion status (transfusion status cohort; *n* = 233). GP: general practitioner; MF: myelofibrosis; TD: transfusion-dependent; TI: transfusion-independent, TR: transfusion-requiring

Among the 233 patients in the transfusion status cohort, mean numbers of all-cause and MF-specific hospitalizations during follow-up were lowest for TI patients (all-cause, 3.8; MF-specific, 0.3) and highest for TD patients (all-cause, 5.8; MF-specific, 1.5; Fig. 6A).

Compared with TI and TR patients, TD patients visited specialists more often (mean number of visits, 25.8 [Fig. 6B]). Two-thirds of patients in the TD and TR cohorts required nursing care (64.1% and 66.1%, respectively) compared with 43.3% of the TI group. To note, this analysis reflects only the absence or presence of nursing care and not the intensity of nursing care.

The mean number of outpatient and inpatient visits with associated transfusion during follow-up were highest among patients who were TD at the landmark date (14.7 and 3.0, respectively) and reduced stepwise with less severe transfusion status (TR, 3.8 and 2.0, respectively; TI, 2.4 and 1.0, respectively; Fig. 6C).

## Discussion

This study was the first to examine real-world treatment sequencing, OS, and HCRU associated with anemia in patients with MF in Germany. It was found that only 41.8% of patients received treatment for MF, and there was limited use of ruxolitinib (the only JAKi available for most of the study period) or allo-HSCT. Indeed, despite being recommended as a 1 L treatment for patients with intermediate- or high-risk disease, < 30% of patients received treatment with ruxolitinib. There was a substantial decrease in patient numbers from 1 L to 2 L (63.8% of patients receiving 1 L treatment did not receive 2 L treatment) and from 2 L to 3 L (71.4% of patients receiving 2 L treatment did not receive 3 L treatment). The median time to ruxolitinib discontinuation of 32.1 months was similar to the amount of time reported in other studies for resistance to ruxolitinib to develop [23, 24]. In addition, there was a high burden for supportive anemia treatments. Overall, 69.0% of patients received at least one anemia treatment throughout the follow-up period, and this treatment was typically required within 2 months of MF diagnosis, and before MF treatment was initiated. Most commonly, anemia was managed with blood cell transfusions (received by 59.8% of patients). Possible reasons for the low use of MF treatment include patient frailty, low-risk disease, early mortality or rapid clinical deterioration and labelling restrictions of JAKis for pre-fibrotic MF.

In our study, among the 233 patients with anemia included in the transfusion cohort, 27.5% were classed as TD. Among patients who became TD, progression occurred rapidly, within a median of 3.5 months, highlighting the need for treatments that could provide better prevention of anemia in MF. The rate of conversion to transfusion dependency in this study is difficult to compare with the literature, given that it applied a landmark period of 180 days during which transfusion dependency was defined.

In the present study, the need for blood transfusions was associated with reduced survival rates in patients with MF. OS for the TD cohort was significantly lower than for the TI cohort, and median OS was numerically shorter than for the non-TD cohort. This aligns with the existing literature, which associates transfusion dependency with inferior survival outcomes in patients with MF [10, 17]. There was no significant difference in OS between the TR and TI cohorts, but the overall trend favored TI patients. There was also a lack of significant difference in OS outcomes between propensity score-matched patients in the TI and non-TI cohorts. However, careful definition of TR status may be important to detect significant differences between TR and TI cohorts [25–27]. These results suggest that, while any transfusion during the landmark period may be a negative prognostic factor for OS, the frequency of transfusions has a crucial role in survival outcomes. However, the study may have underestimated mortality, as patients who died within the 180-day landmark period were excluded. TD patients also consistently had higher HCRU compared with TR and TI patients, which aligns with previous studies in the United States and Italy and emphasizes the HCRU burden associated with transfusion dependence in patients with MF [28, 29]. The higher HCRU among TD patients may be attributed to the greater severity of their MF disease state, reflecting the compounded effects of severe anemia due to advanced disease progression.

In light of the HCRU and survival impact of TD, anemia clearly presents a challenge for the clinical management of patients with MF. Improving the management of MF is particularly important in earlier treatment settings, given that many patients do not progress to later lines. The JAKis ruxolitinib and fedratinib are not able to prevent anemia, and, indeed, can exacerbate this complication [5, 7, 8]. The novel JAKi momelotinib, with its alternative mechanism of action, may have an important clinical role in MF by improving anemia and preventing or reversing red blood cell transfusion dependence [10–13].

This study assessed patients with MF in the German AOK PLUS database from the Saxony and Thuringia regions, inherently limiting the sample to around 4.5% of the German population (as of 2021) and to two German states. However, as healthcare insurance is homogenous across Germany, in accordance with the German legal framework, this cohort may also be representative of German patients with MF outside of the AOK PLUS geographical coverage, and, therefore, provides insight into the clinical and economic landscapes for MF during the study period (2010–2022). German claims databases may have missing data and coding inaccuracies, particularly for outpatient diagnoses. To ensure accuracy, we assumed an outpatient diagnosis to be reliable if a respective diagnosis was documented in two distinct quarters, a standard practice for diagnosis eligibility [30]. Anemia diagnoses may be underreported, as anemia treatment use was used as a proxy. In addition, certain clinically relevant details (e.g., MF risk stratification) were not available, as data were restricted to those relevant to reimbursement. Key parameters relating to baseline MF risk factors (such as mutation status or peripheral blasts), which influence both transfusion dependence and survival outcomes, were not available particularly in the propensity score-matching analysis. Lines of therapy were not documented in the claims data and, therefore, had to be approximated based on prescription dates and an algorithm for treatment discontinuation. Treatment dosages were also not available, so it was not possible to assess whether patients always received adequate dosages. As fedratinib was approved for the treatment of MF in 2021, the availability of this JAKi would have been very limited during the study period, so its use could not be fully analyzed. Similarly, real-world use of momelotinib (approved for clinical use in January 2024) could not be evaluated. Finally, as the study utilized a landmark period for assessing transfusion status, this may have introduced a time bias, and, therefore, OS and HCRU were also assessed from MF diagnosis.

Overall, the current study documents the high clinical and economic burden associated with anemia in patients with MF within Germany, particularly among those who are TD. It also documented a current reliance on blood transfusions to manage this symptom, despite transfusion dependency being associated with worse survival outcomes and higher HCRU. Therefore, this study highlights an unmet need for novel treatments for MF that can better manage or prevent anemia, and for early proactive treatment of anemia in MF to be prioritized. Additional real-world studies will be needed to explore the impact of novel treatments introduced into clinical practice.

## Supplementary Information

Below is the link to the electronic supplementary material.

Supplementary Material 1 (DOCX 300 KB)

## Data Availability

Data used for this publication were curated and analyzed by Cytel using the AOK PLUS health insurance fund database. Requests for access to any of these anonymized subject-level data should be made to AOK PLUS.
