# Supplementary material for: Myelofibrosis and anemia: a German claims data analysis to describe treatment sequencing, survival outcomes, and healthcare resource utilization
Source: Ann Hematol. 2026 Jun 4;105(9):365. doi: 10.1007/s00277-026-07033-w (PMC13451312; doi:10.1007/s00277-026-07033-w)
Supplement: Supplementary file 1 [file 277_2026_7033_MOESM1_ESM.docx]

**Supplementary material**

**Supplementary methods**

**Data source**

In 2021, the AOK PLUS fund covered approximately 3.3 million people, representing 54% of the local population in Saxony and Thuringia and 4.5% of the statutorily insured population in Germany [1,2]. Collected data included demographic information and detailed reimbursement claims for inpatient and outpatient care covered by statutory health insurance.

**Ethical approval**

Because the analysis was based on anonymized, retrospective, and non-interventional data, neither ethical approval nor patient informed consent were required under German national legislation [3]. The use of the data for this study was authorized by the responsible authority, the involved health insurance fund, AOK PLUS. The study protocol was scientifically reviewed and approved by AOK PLUS.

**Study population**

Adult patients (≥18 years of age at index diagnosis) were included if they had at least one inpatient diagnosis of MF (based on International Statistical Classification of Diseases and Related Health Problems, 10th revision, German modification [ICD-10-GM] codes D47.4, D47.1, or C94.6) or two outpatient MF diagnoses in different quarters within 1 year from January 1, 2012, to December 31, 2021. Following the first observed myelofibrosis (MF) diagnosis including the MF index date, eligible patients were required to have at least one inpatient or confirmed outpatient diagnosis of osteomyelofibrosis or at least one prescription or inpatient administration of a Janus kinase inhibitor (JAKi) (ruxolitinib/fedratinib), based on Anatomical Therapeutic Chemical (ATC) classification system and Operationen-und Prozedurenschlüssel/Operation and Procedure Classification System (OPS) codes. In addition, patients were required to have been continuously enrolled with the health insurance fund for at least 365 days prior to index diagnosis. Patients were excluded if they had a prior MF diagnosis before the MF index date or had a diagnosis of essential thrombocythemia (ET) or polycythemia vera (PV) after the index diagnosis. See also Table S1.

**Analysis cohorts**

The treatment sequencing cohort comprised all patients meeting the main eligibility criteria detailed above. The overall survival (OS)/ healthcare resource utilization (HCRU) descriptive cohort and transfusion status cohort excluded patients if they had an allogeneic hematopoietic stem cell transplantation (allo-HSCT) or splenectomy procedure after the index date. To be included in the transfusion status cohort, patients had to have at least one prescription or inpatient administration of a treatment for anemia (as listed in Table S2) from 100 days before the MF index date to end of follow-up. They also needed to have at least 180 days of continuous enrollment within the health insurance fund following the first observed anemia treatment date. Patients with a diagnosis of acute myeloid leukemia (AML) between the MF index date and 180 days after the first anemia diagnosis were excluded from this cohort. In this cohort, the anemia index date was the date of the first anemia treatment and the landmark date was the last day of the landmark period.

**Landmark period**

The 180-day landmark period was selected to provide sufficient time after initiation of anemia treatment to observe transfusion patterns and reliably classify patients. In real-world practice, transfusions may occur intermittently and not immediately after treatment initiation. A 6-month window allows adequate time for patients to meet the transfusion dependency criterion (≥3 transfusions within 12 weeks).

**Assessments**

Treatments evaluated included MF-specific treatments (JAKis, peginterferon alpha-2a, immunomodulators, hydroxycarbamide, and allo-HSCT) as well as concomitant treatments (anemia treatments, including transfusion and splenectomy). Table S2 lists the treatment identification codes relevant for each category of MF and concomitant treatment. Definitions used within the study are summarized in Table S3.

HCRU included all-cause and MF-specific hospitalizations, specialist/general practitioner visits, provision of any nursing care, and outpatient/inpatient visits associated with transfusions.

**Statistical analysis**

Patient demographics, comorbidities, treatment sequencing, and HCRU events were summarized using descriptive statistics. Treatment sequencing was assessed from the MF index date until the end of follow-up, and treatment lines were illustrated in a Sankey diagram. Time to treatment initiation and time to switch/discontinuation were described using the Kaplan–Meier method. Survival and HCRU outcomes were assessed from the MF index date or landmark date until the end of follow-up (study end, end of insurance, AML transformation, or death). Kaplan–Meier estimates for OS were depicted with survival curves, and OS hazard ratios were calculated using multivariate Cox proportional hazards regression.

Propensity-score matching was used to match non-TI and TI patients for OS comparison using a 1:2 nearest-neighbor matching algorithm with replacement. Covariates included in this matching included age, sex, time since MF diagnosis, landmark date year, diagnosis of thrombotic disease or thrombocytopenia, and treatment with a JAKi.

**Table S1** Eligibility criteria

| **Analysis** | **Eligibility criteria** | **Identification codes** |
| --- | --- | --- |
| All **(treatment sequencing cohort)** | **Inclusion**   - At least one inpatient or two confirmed outpatient diagnoses in two different quarters within 12 months for MF from January 1, 2012, to December 31, 2021 (first diagnosis date = MF index date) - Following the first observed MF diagnosis (including the MF index date):   - At least one inpatient or confirmed outpatient diagnosis of osteomyelofibrosis, **or**   - At least one prescription or inpatient administration of a JAKi medication - ≥18 years of age at MF index date - ≥365 days of continuous enrollment prior to MF index date   **Exclusion**  ≥1 MF diagnosis any time prior to the MF index date  ≥1 ET/PV diagnosis at any time following the MF index date | - MF:   - Osteomyelofibrosis: ICD-10-GM D47.4   - Chronic myeloproliferative disease: ICD-10-GM D47.1   - Myelodysplastic and myeloproliferative disease, not elsewhere classified: ICD-10-GM C94.6 - JAKi:   - Ruxolitinib: ATC L01XE18 (prior to 2021), L01EJ01 (since 2021), OPS 6-009.4   - Fedratinib: ATC L01EJ02 - ET: ICD-10-GM D47.3 - PV: ICD-10-GM D45 |
| **Additional eligibility criteria** | | |
| OS and HCRU analysis from MF index date **(OS/HCRU descriptive cohort)** | **Exclusion**  ≥1 allo-HSCT or splenectomy (total or partial) procedure at any time after first observed MF diagnosis (inclusive) | - Allo-HSCT: OPS 5-411.2-5-411.5, 8-805.2-8-805.5, 8-863 - Splenectomy: OPS 5-413 |
| OS and HCRU analysis from landmark date of TI, TR, and TD patients with MF **(transfusion status cohort)** | **Inclusion**   - ≥1 prescription or inpatient administration of an anemia treatment from 100 days before MF index date to end of follow-up (anemia index date = date of first anemia treatment)^a^ - ≥180 days of continuous enrollment following the anemia index date (date of first anemia treatment)   **Exclusion**   - ≥1 allo-HSCT or splenectomy (total or partial) procedure at any time after first observed MF diagnosis (inclusive) - ≥1 AML diagnosis following the MF index date and before the landmark date (inclusive)^b^ | - Anemia treatment:   - Erythropoietin: ATC B03XA01   - Androgens: ATC G03B   - Blood transfusion: EBM 02110, 02111, 02112, OPS 8-800.0, 8-800.1, OPS 8-800.c   - Corticosteroids: ATC H02 & anemia diagnosis - Anemia: ICD-10-GM D46.0, D46.1, D46.2, D46.4, D46.5, D50.- (excl. D50.1), D51.- (excl. D51.2), D52.-, D53.-, D55.-, D58.8, D58.9, D59.0, D59.1, D59.2, D59.4, D59.8, D59.9, D61.-, D62.-, D63.0, D63.8, D64.- - Allo-HSCT: OPS 5-411.2-5-411.5, 8-805.2-8-805.5, 8-863 - Splenectomy: OPS 5-413 - AML: ICD-10-GM C92.0, C92.4, C92.5, C92.6, C92.8 |

^a^Only for cases in which patients only receive corticosteroids and no other pre-specified anemia treatment, patients must have had at least one inpatient or confirmed diagnosis code for anemia (date of first corticosteroid prescription in anemia finding window = anemia index date). If the first anemia treatment was received prior to the MF index date, the anemia index date was set equal to the MF index date

^b^A landmark period was used to assess the transfusion status of patients (180-day period starting from the anemia index date). The landmark date was the last day of the landmark period

allo-HSCT: allogeneic hematopoietic stem cell transplantation; AML: acute myeloid leukemia; ATC: Anatomical Therapeutic Chemical; EBM: Einheitlicher Bewertungsmaßstab [German uniform evaluation standard]; ET: essential thrombocythemia; HCRU: healthcare resource utilization; ICD-10-GM: International Statistical Classification of Diseases and Related Health Problems, 10th revision, German modification; JAKi: Janus kinase inhibitor; MF: myelofibrosis; N/A: not applicable; OPS: Operationen-und Prozedurenschlüssel [operation and procedure codes]; OS: overall survival; PV: polycythemia vera; TD: transfusion-dependent; TI: transfusion-independent, TR: transfusion-requiring

**Table S2** Identification codes for MF treatments and concomitant treatments

| **MF or concomitant therapy** | **Category** | **Treatment** | **Identification** |
| --- | --- | --- | --- |
| MF therapy | Interferons | Peginterferon alpha-2a | ATC L03AB11, L03AB60 (combinations) |
|  | JAK-inhibitors | Fedratinib | L01EJ02 |
|  |  | Ruxolitinib | ATC L01XE18 (prior to 2021), L01EJ01 (since 2021), OPS 6-009.4 |
|  | Allo-HSCT | Allogeneic transplantation of hematopoietic stem cells from the bone marrow | OPS 5-411.2–5-411.5 |
|  |  | Transfusion of peripherally obtained hematopoietic stem cells, allogeneic | OPS 8-805.2–8-805.5 |
|  |  | Allogeneic hematopoietic stem cell therapy | OPS 8-863 |
|  | Immunomodulators | Thalidomide | ATC L04AX02 |
|  |  | Lenalidomide | ATC L04AX04, OPS 6-003.g |
|  |  | Pomalidomide | ATC L04AX06, OPS 6-007.a |
|  | Antineoplastic agents | Hydroxycarbamide (hydroxyurea) | ATC L01XX05 |
| Concomitant therapy | Anemia treatment | Erythropoietin | ATC B03XA01 |
|  |  | Androgens | ATC G03B |
|  |  | Blood transfusion | General blood transfusion: EBM 02110, 02111, 02112 Whole blood transfusion: OPS 8-800.0, 8-800.1 RBC transfusions (erythrocyte concentrate): OPS 8-800.c |
|  |  | Corticosteroids^a^ | ATC H02 |
|  | Surgery | Splenectomy | OPS 5-413 |

^a^Corticosteroids were only included as an anemia treatment in a sensitivity analysis

allo-HSCT: allogeneic hematopoietic stem cell transplantation; ATC: Anatomical Therapeutic Chemical; EBM: Einheitlicher Bewertungsmaßstab [German uniform evaluation standard]; JAK: Janus kinase; MF: myelofibrosis; OPS: Operationen-und Prozedurenschlüssel [operation and procedure codes]

**Table S3** Definitions used within the study

| **Parameter** | **Definition** |
| --- | --- |
| Defined daily dose | Technical unit of measurement: The assumed average maintenance dose per day for a drug used for its main indication in adults (defined by the WHO and adjusted for Germany by the scientific institute of AOK PLUS) |
| Medication supply | Days covered by a prescription are calculated based on the prescribed pack size (dosage, number of tablets) and the defined daily dose |
| Stockpiling | The medication stock accumulates if an additional medication dispensation was observed before the end of supply of the preceding prescription |
| Line of treatment | Sequence of treatments following the index treatment (1L, 2L, 3L) |
| Treatment switch | A switch indicates the beginning of a subsequent line of therapy. Only MF treatments were considered for the definition of therapy lines   - Outpatient setting: Date of first prescription of an agent not part of the previous treatment line (prescription of a new agent >28 days after the start of the previous line) - Inpatient setting: Date of hospital admission if an agent that was not part of the previous treatment line was given during the respective hospitalization (hospital admission >28 days after the start of the previous line) |
| Treatment discontinuation | Supply gap (no prescription/inpatient admission of any of the agents part of the current therapy line) following the end of supply of an outpatient prescription   - JAKi-containing regimens: The date of discontinuation was the date of the last observed prescription + twice the days covered by the respective medication supply - Non-JAKi-containing regimens: The date of discontinuation was 90 days after the end of supply - Hospital was considered covered by the respective medication - Stockpiling was assumed and remaining stock was considered after hospital discharge - If the same treatment was resumed after treatment discontinuation, this was reported as a subsequent line of therapy |
| Line start date | 1L: Date of the first prescription of any relevant MF-related treatments or date of hospital admission if a relevant agent was administered during the hospital stay (inpatient setting) with no prescription or administration of any MF-related treatments occurring prior  2L/3L: Date of treatment switch or date of prescription of an agent following treatment discontinuation (outpatient setting) or date of hospital admission if a relevant agent was administered during the hospital stay following treatment discontinuation (inpatient setting) |
| Line end date | One of the following: Date of treatment switch, treatment discontinuation, end of study period, end of continuous insurance or death, whichever occurred first |
| Combination therapy | Defined as all relevant agents prescribed within a treatment window of 28 days following the line start date |

1L: first-line; 2L: second-line; 3L: third-line; JAKi: Janus kinase inhibitor; MF: myelofibrosis; WHO: World Health Organization.

**Table S4** Demographics and baseline characteristics of all analyzed cohorts

| **Characteristic** |  | **Treatment sequencing cohort (*n* = 555)** | **OS/HCRU descriptive  cohort  (*n* = 510)** | **Transfusion status  cohort  (*n* = 233)** | **Transfusion status cohort after propensity matching^a^** | | |
| --- | --- | --- | --- | --- | --- | --- | --- |
|  |  |  |  |  | **Overall (*n* = 346)** | **TI  (*n* = 173)** | **Non-TI  (*n* = 173)** |
| **Patient demographics** |  |  |  |  |  |  |  |
| Age at landmark date, mean (SD) |  | 70.1 (13.5)^b^ | 71.0 (13.5)^b^ | 73.3 (12.1) | 75.1 (10.7) | 75.6 (9.7) | 74.5 (11.5) |
| Sex, male, *n* (%) |  | 312 (56.2) | 281 (55.1) | 135 (57.9) | 191.5 (55.3) | 95.5 (55.2) | 96.0 (55.5) |
| Landmark date year, *n* (%) |  |  |  |  |  |  |  |
| 2012–2014 |  | 167 (30.1) | 157 (30.8) | 60 (25.8) | 103 (29.8) | 54 (31.2) | 49 (28.3) |
| 2015–2017 |  | 181 (32.6) | 163 (32.0) | 64 (27.5) | 100 (28.9) | 51 (29.5) | 49 (28.3) |
| 2018–2020 |  | 164 (29.5) | 152 (29.8) | 73 (31.3) | 103 (29.8) | 49 (28.3) | 54 (31.2) |
| 2021–2022 |  | 43 (7.7) | 38 (7.5) | 36 (15.5) | 40 (11.6) | 19 (11.0) | 21 (12.1) |
| **Comorbidities (365 days prior to landmark date to 1 day prior to landmark date)** | | | |  |  |  |  |
| CCI score, mean (SD)^c^ |  | 3.4 (2.9) | 3.5 (2.9) | 5.3 (3.3) | 5.3 (3.0) | 5.2 (0.0) | 5.5 (0.0) |
| CCI score category, *n* (%)^c^ |  |  |  |  |  |  |  |
| 0–1 |  | 157 (28.3) | 141 (27.6) | 31 (13.3) | 43.5 (12.6) | 24.5 (14.2) | 19 (11.0) |
| 2–3 |  | 176 (31.7) | 161 (31.6) | 52 (22.3) | 58.5 (16.9) | 21.5 (12.4) | 37 (21.4) |
| 4–5 |  | 91 (16.4) | 86 (16.9) | 47 (20.2) | 81 (23.4) | 44 (25.4) | 37 (21.4) |
| 6+ |  | 127 (22.9) | 119 (23.3) | 103 (44.2) | 163 (47.1) | 83 (48.0) | 80 (46.2) |
| Anemia diagnosis, *n* (%) |  | 254 (45.8) | 233 (45.7) | 203 (87.1) | 264 (76.3) | 98 (56.6) | 166 (96.0) |
| Thrombocytopenia, *n* (%) |  | 91 (16.4) | 81 (15.9) | 70 (30.0) | 115.5 (33.4) | 56.5 (32.7) | 59 (34.1) |
| ET, *n* (%) |  | 14 (2.5) | 13 (2.5) | 2 (0.9) | 2 (0.6) | 0 (0.0) | 2 (1.2) |
| PV, *n* (%) |  | 4 (0.7) | 4 (0.8) | 0 (0.0) | 0 (0.0) | 0 (0.0) | 0 (0.0) |
| **Baseline HCRU (365 days prior to landmark date to 1 day prior to landmark date)** | | | |  |  |  |  |
| All-cause hospitalizations, *n* (%) |  | 318 (57.3) | 291 (62.4) | 210 (90.1) | 287 (82.8) | 128 (73.7) | 158 (86.7) |
| Number of events, mean (SD) |  | 1.2 (1.8) | 2.1 (1.8) | 4.9 (5.8) | 2.7 (2.9) | 1.8 (1.7) | 5.3 (6.3) |
| Oncologist/hematologist visits, *n* (%) |  | 126 (22.7) | 115 (24.7) | 146 (62.7) | 223 (64.5) | 115 (66.5) | 108 (62.4) |
| Number of events, mean (SD) |  | 1.7 (6.6) | 7.6 (12.5) | 6.7 (9.4) | 6.2 (8.5) | 5.7 (7.4) | 6.8 (9.4) |
| **Follow-up (landmark date to end of follow-up or censor)** | | | |  |  |  |  |
| Follow-up time (months), mean (SD) |  | 38.5 (32.3) | 39.0 (32.2) | 29.9 (26.3) | 32.4 (27.2) | 36.6 (28.4) | 28.3 (25.5) |
| AML transformation during follow-up, *n* (%) |  | 67 (12.1) | 46 (9.0) | 10 (4.3) | 10 (2.9) | 0 (0.0) | 10 (5.8) |

^a^Propensity-score matching was performed with 1:2 matching of non-TI to TI patients. Weights were assigned to the TI group to adjust for the fact that each non-TI patient was matched with two TI patients

^b^Age at MF index date

^c^CCI unadjusted for age

AML: acute myeloid leukemia; CCI: Charlson Comorbidity Index; ET: essential thrombocythemia; HCRU: healthcare resource utilization; MF: myelofibrosis; OS: overall survival; PV: polycythemia vera; SD: standard deviation; TI: transfusion-independent

**Fig. S1.** Study attrition.

allo-HSCT: allogeneic hematopoietic stem cell transplantation; AML: acute myeloid leukemia; ET: essential thrombocythemia; HCRU: healthcare resource utilization; JAKi: Janus kinase inhibitor; MF: myelofibrosis; OS: overall survival; PV: polycythemia vera.

# Supplementary references

1.	AOK Plus AOK Plus contribution, benefits and service. <https://www.health-insurance.de/statutory-health-insurance/list/245-AOK-PLUS.html>. Accessed 6 May 2025

2.	Slowley A, Weinmann S, d'Estrube T, Phiri K, Karl FM, Gleißner E, Mueller S, Junker S, Göthert JR (2024) Myelofibrosis and anemia: A German claims data analysis to describe epidemiology and current treatment. Eur J Haematol 113 (5):704-715. doi:10.1111/ejh.14284

3.	Federal Ministry of Justice and Consumer Protection Book Ten of the Social Code - Social Administrative Procedures and Social Data Protection - (SGB X). Section 75 Transmission of Social Data for Research and Planning. <https://www.gesetze-im-internet.de/sgb_10/__75.html>. Accessed 13 May 2025
